# Cholesterol Granuloma: An Unusual Presentation Within the Mandible of a Pediatric Patient

**DOI:** 10.7759/cureus.24163

**Published:** 2022-04-15

**Authors:** Joseph M Gosnell, Hisham Marwan, Suimin Qiu

**Affiliations:** 1 Pathology, University of Texas Medical Branch at Galveston, Galveston, USA; 2 Oral and Maxillofacial Surgery, University of Texas Medical Branch at Galveston, Galveston, USA

**Keywords:** mandibular marsupialization, pediatric, mandible, dentigerous cyst, odontogenic cyst, marsupialization, cholesterol granuloma

## Abstract

Cholesterol granulomas, while a common pathological finding, are rarely reported within the mandible. Herein, we report the case of a pediatric patient who presented with a cholesterol granuloma within a periapical cyst, extending from the inferior aspect of tooth #31 to the mandibular condyle, with no prior history of infection or trauma to the region and underwent successful marsupialization of the lesion.

## Introduction

Cholesterol granulomas form as the result of a foreign body reaction to precipitated cholesterol crystals within an area of tissue where previous bleeding occurred. While the pathogenesis of this bleeding is still debated [1-2], the result is a mix of cholesterol clefts, hemosiderin pigment, and multi-nucleated giant cells with no epithelial lining. There are few reports of cholesterol granuloma occurring in the mandible. After a comprehensive literature review, our team was able to identify 13 cases of mandibular cholesterol granulomas. In the current case, we detail an extremely rare presentation of this entity, as it is located not only within the mandible but also occurred in a pediatric patient with no history of trauma or infection in the surrounding tissue. Furthermore, this is the first report in the literature that describes marsupialization as a treatment for this entity.

## Case presentation

**Figure 1:**
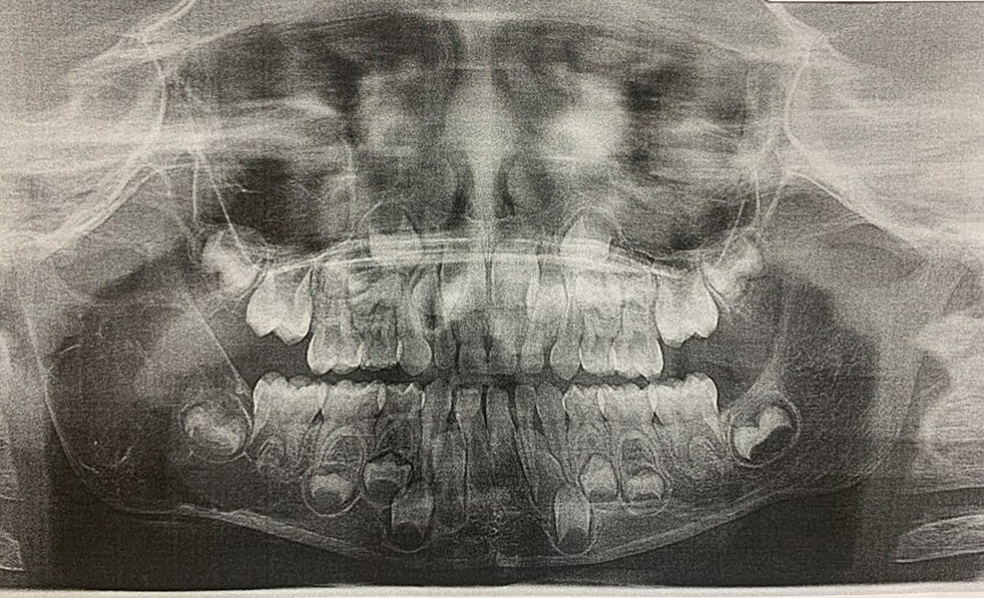
Panoramic radiograph with mandibular lesion

We report a case of a six-year-old girl who presented to the Oral and Maxillofacial surgery clinics at the University of Texas Medical Branch with swelling of the right posterior mandible and panoramic radiograph (Figure 1). The swelling was discovered during a routine annual pediatric evaluation, and she was referred to Oral and Maxillofacial Surgery clinics for further management. Cone-beam computed tomography (CT) scan showed a large, multiloculated lesion associated with tooth bud # 31, extending from the body of the mandible to the head of the right mandibular condyle. The patient underwent a biopsy to differentiate between a mass, cystic lesion, and arteriovenous malformation. During the procedure, a very thin lining was identified within a small area of the lesion, no mass was identified, and arteriovenous malformation (AVM) was ruled out. During the biopsy procedure, the lesion was deemed to be non-tumorous, and due to the enormous size of the lesion, the surgical team decided to marsupialize the lesion in an attempt to reduce its size before the enucleation and curettage surgery.

**Figure 2:**
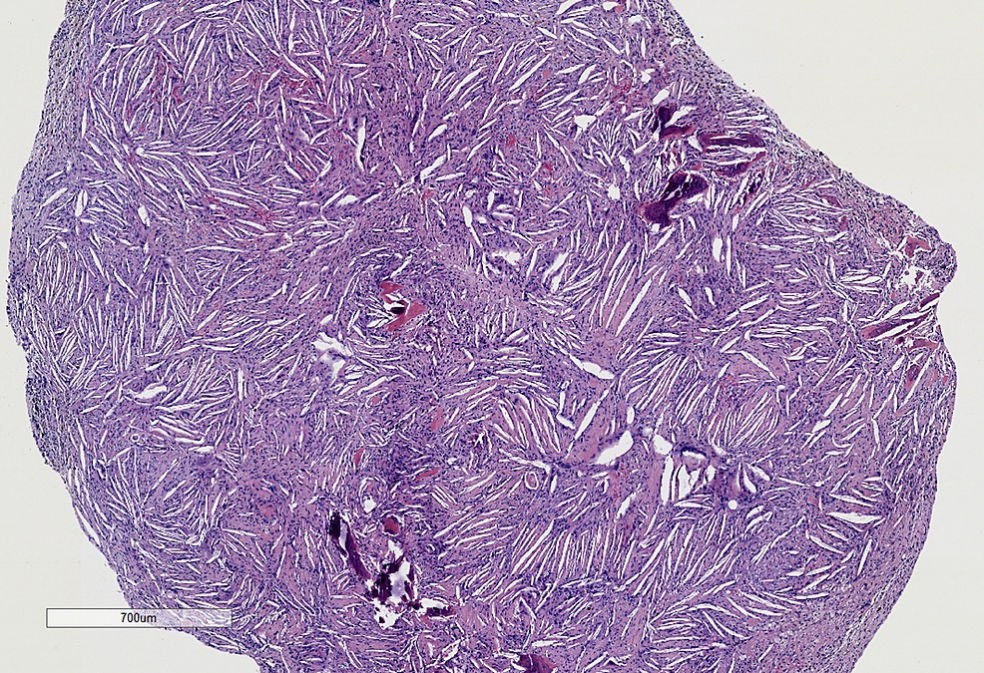
Cholesterol granuloma at 20X magnification

**Figure 3:**
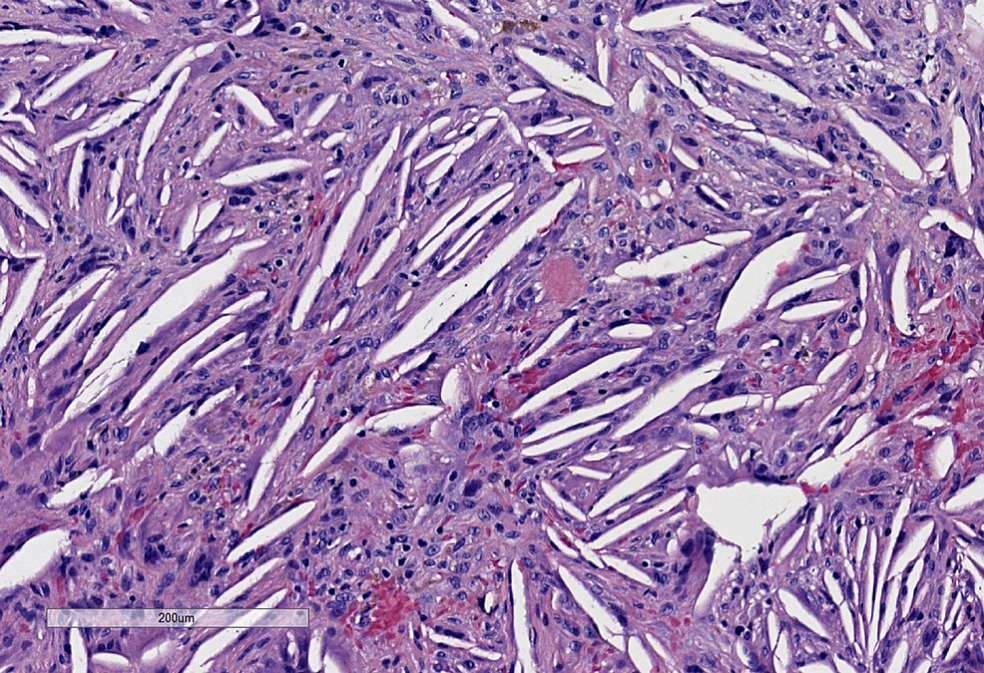
Cholesterol clefts, foamy macrophages, hemosiderin pigment, and multinucleated giant cells at 100X magnification

The lining was sent for pathological examination. Microscopic examination revealed granulation tissue with abundant cholesterol clefts, hemosiderin pigment, foamy macrophages, and multinucleated giant cells (Figures 2-3).

It is not uncommon that a cholesterol granuloma will form within a dentigerous cyst [3] or similar structure in response to an inflammatory insult, such as a tooth root infection; however, in this instance, the incomplete lining of the lesion was not obtained during the biopsy. The lack of both previous insults, mechanical or microbiological, marks this case as unusual, as nearly all previously reported cases had indications of prior trauma or infection associated with the region in which the cholesterol granuloma formed. Additionally, the lack of epithelial lining most likely points toward a periapical granuloma as the lesion the cholesterol granuloma formed within.

**Figure 4:**
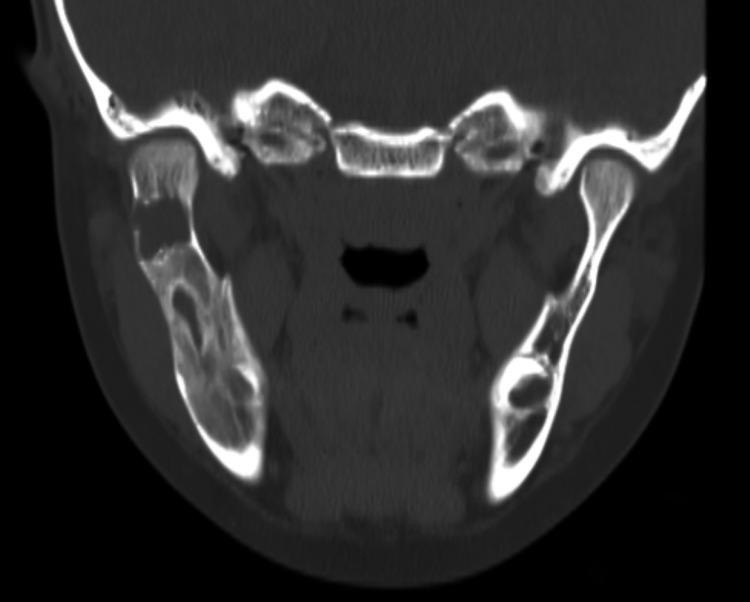
Post-marsupialization CT of the mandible

The patient tolerated the marsupialization very well for eight weeks. Follow-up CT maxillofacial showed a significant reduction in the size with excellent bone regeneration (Figure 4).

Currently, the patient is scheduled for removal of the remaining lesion via an intraoral approach.

## Discussion

A review of the literature returned only 13 previously reported cases of cholesterol granulomas that presented within the mandible or jaw (Table 1) [4-8]. This case report provides more information about this rare pathological entity. Additionally, it shows that cholesterol granuloma of the jaws can affect children, and marsupialization is an option for the treatment.

**Table 1:** Previously reported mandibular cholesterol granuloma cases in the literature

Authors	Cases	Publication Year	Location	Patient Age	Reference
Saruhan et al.	1	2018	Mandible	58 Yrs	4
Fernandez-Olarte et al.	1	2017	Mandible	31 Yrs	2
Kamboj et al.	3	2016	Mandible	45, 38, 47 Yrs	3
Alkan et al.	2	2014	Mandible	57, 67 Yrs	6
Lee et al.	1	2010	Mandible	68 Yrs	7
Shin et al.	3	2007	Mandible	68, 47, 19 Yrs	12
Kim et al.	1	2007	Mandible	68 Yrs.	5
Hirschberg et al.	1	1988	Mandible	55 Yrs	8

While the vast majority of cholesterol granulomas present in the middle ear, the petrous apex, or the mastoid, this patient was found to have a large, expansile, multi-loculated radiolucent lesion of the right posterior mandible, inferior to the right lower second molar (tooth #31), that extended to the mandibular condyle. This lesion can present less commonly in the mediastinum, frontal sinuses, and tooth-associated structures [9] and in the kidneys, brain, testicles, ovaries, and other soft-tissue structures.

There are two major theories of pathogenesis for cholesterol granulomas: 1. Exposed marrow theory: hyperplastic mucosa invades underlying bone and exposes marrow, which consequently bleeds [10]; and 2. Obstruction-vacuum theory: lack of drainage leading to repeated episodes of edema and eventually bleeding [11]. Both theories are controversial, but the obstruction-vacuum theory is not a good fit for presentation within the mandible [12]. The formation of an odontogenic cyst or similar lesion may lead to exposed bone marrow, resulting in a cholesterol granuloma.

Important differential diagnoses to consider when observing radiolucent and mixed-density lesions within the mandible include odontogenic keratocyst, mandibular hemangioma, aneurysmal bone cyst, mandibular AVM, central giant cell granuloma, Ewing sarcoma, juvenile ossifying fibroma, osteosarcoma, or Brown's tumor. Relying on previous imaging characteristics can be extremely helpful in narrowing down the differential diagnosis and informing potential diagnostic and therapeutic strategies.

## Conclusions

It is important to consider cholesterol granuloma in the differential diagnosis of mandibular cystic lesions with mural or solid components, including those which present in pediatric patients.

To the best of our knowledge, this is the only case report of pediatric cholesterol granuloma of the mandible, as well as the first to be successfully treated with marsupialization. This report showed the efficacy of this treatment modality in reducing the size of the lesion and minimizing the morbidity of the definitive surgery.
